# Oral immune therapy: targeting the systemic immune system via the gut immune system for the treatment of inflammatory bowel disease

**DOI:** 10.1038/cti.2015.47

**Published:** 2016-01-29

**Authors:** Yaron Ilan

**Affiliations:** 1 Gastroenterology and Liver Units, Department of Medicine, Hebrew University-Hadassah Medical Center, Jerusalem, Israel

## Abstract

Inflammatory bowel diseases (IBD) are associated with an altered systemic immune response leading to inflammation-mediated damage to the gut and other organs. Oral immune therapy is a method of systemic immune modulation via alteration of the gut immune system. It uses the inherit ability of the innate system of the gut to redirect the systemic innate and adaptive immune responses. Oral immune therapy is an attractive clinical approach to treat autoimmune and inflammatory disorders. It can induce immune modulation without immune suppression, has minimal toxicity and is easily administered. Targeting the systemic immune system via the gut immune system can serve as an attractive novel therapeutic method for IBD. This review summarizes the current data and discusses several examples of oral immune therapeutic methods for using the gut immune system to generate signals to reset systemic immunity as a treatment for IBD.

Interactions between susceptibility genes, the environment, the gut microbiome and the systemic immune system have a role in the pathogenesis of inflammatory bowel disease (IBD).^1^ Currently available treatments for IBD, which target the systemic immune system, induce immunosuppression, thereby exposing the patient to the risk of infections and malignancy. The interplay between the gut and the systemic immune system determines the final effect on target organs, including the bowel mucosa. Therefore, the gut immune system was suggested as a potential target for immune modulatory agents that act locally at the level of the bowel as a means for altering the systemic immune response. Oral immune therapy is a method for altering the systemic immune system via an effect on the gut immune system to generate a signal that will affect the systemic immune system. This method does not involve generalized immune suppression. This review summarizes several oral immune modulatory methods that can be used to alter systemic immunity as a means of treatment of IBD.

## Oral immunomodulation to target the systemic immune system

Oral immune therapy is a method for altering the systemic immune system via an effect on the gut immune system. It is based on an inherent mechanism in which the gut immune system inhibits or promotes its reactions towards orally administered antigens.^2, 3^ The capability of the gut mucosa-associated immune system to mount an immune response against pathogenic antigens, while maintaining ignorance or active suppression against non-pathogenic antigens undines this phenomenon. It is associated with the ability of the innate immune system of the gut to generate signals that promote systemic adaptive responses. Oral immune therapy uses a physiological system that responds to food or pathogenic antigens, gut microbiome-derived epitopes, or any other type of adjuvants or orally administered antigens to which the gut mucosa is exposed.^4^ Oral tolerance can be viewed as one category of oral immune therapy, and is defined as a specific suppression of humoral and/or cellular immune responses to an antigen, by the administration of the same antigen, or towards bystander orally administered epitopes.^5^

Oral immune therapy is a valid approach to prevent and treat unwanted immune responses that cause a variety of diseases or that complicate the treatment of a disease, and can be used for the treatment of immune-mediated or immune-related disorders.^6^

The mechanism of action of oral immune therapy is not fully elucidated. For oral tolerance, low doses of orally administered antigens favor active suppression with the generation of regulatory T cells (Tregs), whereas high doses favor clonal anergy/deletion.^7, 8, 9^ For other methods of oral immune therapy, promotion of Tregs is one potential mechanism for the suppression of systemic inflammation at target organs via an effect on gut mucosal surfaces.^10^ Crosstalk between antigen-presenting cells in the gut, including dendritic cells (DCs) and T cells, has a role in the generation of the immune signals between the gut and the systemic immune systems. Oral immune therapy is not necessarily antigen specific and can suppress inflammation at the site of inflammation via the induction of suppressor cells, or Tregs, in an antigen-independent manner.^11, 12^ The non-antigen specificity of oral immune therapy may be associated with a bystander effect at the level of the gut or in target organs in which the disease-associated antigen is being presented.^8, 13, 14^ Adjuvants in the gut have an important role in oral immune therapy.^12^ These are critical for appropriate activation of the innate immune system in the gut, thereby affecting the type of signal being delivered to the systemic immune system.^15^

Oral immune therapy has several clinical advantages (Table 1). It uses the inherent ability of the gut immune system to control unwanted systemic immune responses and as such is not associated with generalized immune suppression. It preferentially induces Tregs. It can promote systemic tolerance in an antigen-dependent or -independent manner. In most cases, the compounds used for oral immune therapy do not reach the blood, making this method nontoxic and with minimal side effects. It is not associated with the harmful cytokine release syndrome that is noted for some of the intravenously administered immunomodulatory agents. Oral immune therapy is effective both for preventive therapy and for treatment at the peak of disease. In most cases, as no systemic absorption is required, a relatively low dose is sufficient for a clinically meaningful effect. Oral immune therapy provides a platform that can be used for many disorders. For the patients, it is easily tolerated, and eliminates safety concerns and pain related to needles. Trained medical personnel are not required to administer these drugs, and they are inexpensive.

In contrast, most immune modulatory agents have significant side effects and are associated with some type of generalized immune suppression and an increased risk of infection and malignancy (Table 1). Relatively higher dosages of these drugs are required for the induction of immune suppression. In most cases, treatments are effective only after the onset of the disease, and due to the potential side effects, they cannot be used as preventive measures. Their toxicity and side effects limit their use in a large proportion of patients. Safety concerns and pain related to use of needles limit their use in some patients, and the requirement for trained medical personnel for administration may limit their use in some settings.

Most importantly, the immune modulatory agents used today for IBD do not achieve remission in many patients.^16, 17^ Not all IBD patients benefit from currently available drugs.^18^ Young people with IBD do not want to be on long-term drug therapy. Oral immune therapy, while not yet studied in large cohorts of patients, may provide an answer to this unmet need.

## The interplay between the gut immune system and the systemic immune system: transfer of signals from the bowel is relevant for the pathogenesis of IBD

The gut immune system generates immune signals that can alter the systemic immune response. A complex interplay between many distinct intestinal immune cell types occurs at the gut level, affecting the interplay with the systemic immune system. Several of these components either generate or serve as signals, which alter the response of the systemic immune system.^19^ The immune gastrointestinal barrier is designed to distinguish between beneficial and harmful components in the gut to maintain systemic immune tolerance.^1^ It is composed of intestinal epithelial cells (IECs); cells of the innate immune system, including macrophages, monocytes, neutrophils and DCs; and cells of the adaptive immune system, including T and B lymphocytes and their secreted mediators, the cytokines and chemokines.^1^ Organized lymphoid structures and mucosal cells in the gut wall and beneath the epithelium, and the interaction of many types of cells, including DCs, natural killer T (NKT) cells, M cells, Paneth cells, mast cells, goblet cells and columnar epithelial cells, take part in the gut–systemic immune system interaction.^20, 21^ Secondary lymphoid tissue, such as Peyer's patches, and tertiary lymphoid tissue (the lymphoid follicles) respond to antigenic stimuli by releasing cytokines or producing secretory IgA.^22^ The IECs are in close cooperation with intraepithelial lymphocytes and possess Toll-like receptors on their surface and Nod-like receptors, which sense pathogens or pathogen-associated molecular patterns. All of these components of the gut immune system take part in the interplay with the systemic immune system.

**Figure 1:**
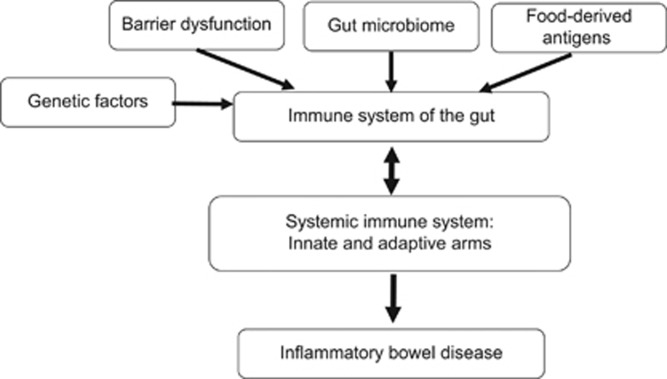
Schematic description of the interaction between the gut and systemic immune systems in the pathogenesis of inflammatory bowel disease.

Components of the mucosal immune system are implicated in the pathogenesis of IBD affecting both the innate and adaptive arms of the systemic immune system.^23^ Disruption of mucosal homeostasis can alter the systemic immune response leading to bowel inflammation such as that seen in IBD (Figure 1).^21^ Barrier dysfunction, the gut microbiome, food-derived antigens and adjuvants are all relevant for activation of the gut immune system thereby affecting the systemic immune response.^23^ Examples for several components of the gut immune system and their role in the pathogenesis of IBD are described below.

## Mucosal barrier

(i) The IECs constitute the first barrier in the gut against the lumen and are required for the maintenance of barrier integrity. They participate in food degradation and absorption, and have a role in intestinal inflammation.^24^ They translate signals coming from the outside world, and deliver information/signals about the gut lumen to immune cells.^25^ The communication occurs from the epithelial cells to the immune system and also in the opposite direction. By producing antimicrobial peptides, IECs alter the gut microbial community. IECs also respond to cytokines and other mediators of immune cells in the lamina propria.^25^ They interact with DCs and other immune cells to drive tolerogenic responses under the steady state, and they release immune mediators to recruit inflammatory cells and to elicit immunity to infectious agents. Dysregulation within the epithelial layer increases intestinal permeability, alters the interactions between IECs and immune cells in the lamina propria, disturbs the intestinal immune homeostasis and can lead to immune derangement and IBD.^24, 26^

(ii) The mucosal barrier has three major components: the mucus layer, the epithelial glycocalyx and the surface epithelium, whose integrity depends on tight junctions.^27^ Large highly glycosylated gel-forming mucins, MUC2, MUC5 and MUC5AC, are major components of the mucus that covers the intestine and stomach, respectively. The mucus limits the number of bacteria that reach the epithelium and the Peyer's patches.^28^ Goblet cells secrete mucin and mucus components and can prevent the presentation of oral antigens to the immune system. These cells deliver small intestinal luminal material to tolerogenic type DCs in the lamina propria.^28^ In addition to gel-forming mucins, the transmembrane mucins MUC3, MUC12 and MUC17 form the enterocyte glycocalyx, which extends a micrometer out from the brush border. The MUC17 mucin shuttles from a surface to an intracellular vesicle controlling the microbiota.^28^ Epithelial tight junction regulates paracellular trafficking of macromolecules.^29^ It is a multi-protein complex that forms a selective permeable seal between adjacent epithelial cells, creating a border between apical and basolateral membrane domains. Patients with IBD have a weakened mucosal barrier. They demonstrate increased intestinal paracellular permeability and increased intestinal tight junctions disruption. Disruption of the intestinal tight junction barrier followed by permeation of luminal toxic molecules induces a perturbation of the mucosal immune system and inflammation which exacerbates IBD.^30^ These changes also affect the transepithelial transport of macronutrients and micronutrients and the gut microbiome in these patients.^29^

(iii) Peyer's patches are lymphoid structures overlain by the epithelium, in which 5% of the cells are specialized M (microfold) epithelial cells, which are a major portal of entry for bacteria. There are no goblet cells in the dome epithelium, and M cells have a scarce glycocalyx allowing easy microbial interaction.^27^ Peyer's patches are sites of lesions in Crohn's disease (CD), and the 'anti-pancreatic' antibody associated with CD is targeting glycoprotein 2, the receptor for type 1 bacterial fimbrial protein (fimH) on M cells.

## Cellular stress

(iv) Autophagy in the intestine, including macroautophagy and xenophagy, has a role in generating an intestinal immune response and antimicrobial protection in some of the patients.^26^ A dysfunctional autophagic mechanism leads to chronic intestinal inflammation in IBD. Genome-wide association studies have identified roles for numerous autophagy genes in IBD. Mucosal susceptibility or defects in sampling of gut luminal antigens via autophagy and crosstalk between the innate immune system and the microbiota activate the innate immune response mediated by enhanced Toll-like receptor activity.^1, 31, 32^

(v) The maintenance of gut mucosal equilibrium requires a balance between enterocyte loss by apoptosis and the generation of new cells by proliferation from stem cell precursors at the base of the intestinal crypts. Receptors of the innate immune system, including Toll-like receptors 2, 4 and 9 and the intracellular pathogen recognition receptor NOD2/CARD15, are associated with the initiation of enterocyte apoptosis. Induction of enterocyte apoptosis in response to activation of these innate immune receptors has a role in the development of IBD.^33^

## Subsets of cells involved in the immune response

(vi) The intestinal mucosa contains numerous DCs that exert protective immunity to infectious agents or tolerance to innocuous antigens, including food and commensal bacteria.^12, 19, 34^ DCs in the gut actively sample both pathogenic and non-pathogenic antigens, including those derived from the microbiota, followed by migration to secondary lymphoid organs in the gut to activate naive T cells.^19^ DCs in the gut induce gut-homing properties on T cells upon activation, enabling T-cell migration back to intestinal sites. Specialized CD103^+^ intestinal DCs promote the differentiation of Foxp3^+^ Tregs via a retinoic acid-dependent process.^35^ DCs dysfunction contributes to IBD development.^34^ Both gut microbiota and food-derived antigens alter intestinal DCs function, and contribute to a loss of tolerance and to induction and progression of IBD.^19^ In patients with IBD, the tolerance/immunity balance is disturbed, leading to chronic intestinal inflammation driven by aberrant T-cell reactivity to intestinal bacteria.^36^ Tolerogenic DCs act by promoting differentiation and expansion of Tregs that efficiently modulate gut inflammation, and they are disturbed in IBD.^37^

(vii) NKT cells are a subset of non-conventional T cells recognizing endogenous and/or exogenous glycolipid antigens when presented by the major histocompatibility complex class I-like antigen-presenting molecules CD1d.^38^ NKT cells are abundant in the gut immune system. Upon T-cell receptor engagement, gut NKT cells rapidly produce cytokines, thus affecting mucosal immunity.^39^ Mucosal and systemic NKT cell development is under the control of the commensal microbiota.^40^ NKT cells in the bowel recognize microbial lipid antigens presented by CD1d. These cells exhibit effector functions in antimicrobial defense and in the modulation of inflammation in the gut. CD1d controls the composition of the intestinal microbiota via regulation of Paneth cell function.^41^ In animal models of IBD, NKT cells make both protective and pathogenic contributions to disease. In patients with ulcerative colitis and in a mouse model in which both CD1d expression and the frequency of subsets of NKT cells are increased, they promote intestinal inflammation.^42^ Oral immune therapy was reported to be associated with promotion of NKT cells in both animal models and humans.^13, 43, 44, 45, 46, 47^

(viii) Immature myeloid cells, known as myeloid-derived suppressor cells (MDSCs), including neutrophilic and monocytic myeloid cells, are found in inflammatory loci and secondary lymphoid organs in mice with intestinal inflammation and in patients with IBD.^48^ They interact with Th17 cells, and their function is determined by the ER stress.^48^ Their pro-inflammatory or immunosuppressive role in IBD is not well defined.

(ix) Regulatory T cells (Tregs) maintain self-tolerance and control excessive immune responses to foreign antigens.^49^ Tregs inhibit effector cells by several mechanisms, including: promotion of inhibitory cytokines; induction of death by cytokine deprivation or cytolysis; local metabolic perturbation by changes in extracellular nucleotide/nucleoside fluxes; alterations in intracellular signaling molecules, such as cyclic AMP; and inhibition of DCs.^49, 50, 51^ The lamina propria constitutes an effector site that actively influences Tregs-cell function.^52^ Tregs must be in the proximity of their target cells within lymphoid organs and the lamina propria in the intestine.^52^ Foxp3(+) Tregs maintain immune balance in the gut via IL-10- and TGF-β-dependent mechanisms.^53^ Their differentiation and function are modulated by intestinal microbiota.^54, 55^ Inflammation in IBD is mediated by inappropriate production of pro-inflammatory cytokines by CD4^+^ T-effector cells, which are not suppressed by Tregs.^56, 57^ Activation of Tregs inhibits the inflammatory response to commensal bacteria and is central for mucosal tolerance. Loss of this mechanism leads to inappropriate immune reactivity toward commensal organisms, contributing to mucosal inflammation in IBD.^51, 58, 59^

(x) Th17 cells infiltrate the intestine of IBD patients, producing IL-17 and amplifying the inflammatory process. Th17 can be converted into either IFN-γ-producing Th1 cells or Tregs.^60^ Antigen presenting cells mediate differentiation of naive T cells into effector T-helper cells, including Th1, Th2 and Th17, and can alter gut homeostasis leading to IBD.^1, 31, 32^

(xi) Macrophages functions change during infection and inflammation. The intestinal macrophage pool requires continual renewal from circulating blood monocytes, unlike most other tissue macrophages, which derive from primitive precursors that subsequently self-renew.^61, 62, 63^ Macrophages in the gut have a role in Tregs function.^52^ As regulatory cells in the gut, macrophages also have a role in the pathogenesis of IBD.^62^

(xii) The function of both T and B cells is required for proper interplay between the gut and the systemic immune systems. Plasticity of CD4^+^ helper T cells is important for the correct function of the gut immune system.^1, 31, 32^

Taken together, these studies suggest that each of the components of the gut immune system is pertinent for the induction and/or progression of IBD. Most of these subsets of cells are involved directly or indirectly in the signaling between the gut and the systemic immune systems, a process that is relevant for the generation and maintenance of the inflammatory process in IBD. Therefore, oral immune therapy, which affects these types of signals between the gut and the systemic immune systems, may aim at several of these targets.

## The gut microbiome in the interplay between the gut and the systemic immune system in IBD

The gut microbiota is required for proper development of the host and maintenance of intestinal homeostasis.^64^ Continuous exposure of the intestinal mucosa to diverse microorganisms and to food-derived products and metabolites is required for proper function of the gut immune regulatory system.^21^ The gut microbiome is important for the generation of the signals between the gut and the systemic immune systems.^65^ Both positive and negative stimulation by luminal products incite the assembly of inflammasomes involved in maintaining the integrity of the intestinal epithelium and a favorable environment for both the host and the microbiota. Indigenous bacteria stimulate the immune system to protect against commensal and exogenous pathogens.^66^ Gut microbiota, mainly Bacteroidetes and Firmicutes, generate a tolerogenic response via acting on DCs and inhibiting the Th17 pathway.^22^ Bacteroides fragilis leads to the production of anti-inflammatory IL-10 by Tregs and lamina propria macrophages. Fragmented filamentous bacteria promote gut inflammation via the induction of Th17 cells.^22^ Increased gut permeability, bacterial translocation and increased lipopolysaccharide levels have been described in patients with immune-associated disorders.^67, 68, 69^ Intestinal barrier loss alone is insufficient to initiate disease.^70^

Dysbiosis and alterations in the intestinal microbiome are associated with IBD.^32^ Gut microbes can induce and sustain the disease.^71^ The loss of normal tolerance to intestinal microbiota and/or to food or environment-derived antigens leads to mucosal damage.^22^ Both in active and in quiescent disease, the fecal- and mucosal-associated microbiomes show reduced diversity. Various cells from IBD patients show increased susceptibility to bacterial products, including flagellin, pili and lipopolysaccharide.^72^ Mucosa-associated adherent, invasive *Escherichia coli* (*E. coli*), which are pro-inflammatory and resistant to killing by mucosal macrophages, may be associated with the pathogenesis of CD.^73^ Bacteria and their products trigger cytokine expression, including tumor necrosis factor alpha (TNF-α) and IL-8 by macrophages and epithelial cells, respectively, in patients with CD.^72^ Bacteria coated with IgA can induce gut inflammation in patients with IBD.^74^ Intestinal microbiota and its toxic components act on Nod1 and Nod2 receptors leading to defective signaling, which accounts for the development of IBD.^22^ Microbiota can also induce anti-inflammatory or regulatory immunological mechanisms. The balance between these opposing processes is relevant for IBD.^32, 75^ IL-10 from macrophages, T cells and B cells, and TGFβ1 from epithelial cells and other non-lymphoid/myeloid cells, are relevant for the anti-inflammatory pattern. The neutralization of TGFβ1 increases Th1 and Th17 responses in IBD; however, exogenous TGFβ1 does not inhibit inflammation because of a block in intracellular signaling mediated by Smad7.^76^ On the basis of the above, fecal transplantation was developed as a mean for the treatment of IBD.^77^

## The liver–gut axis in IBD

The liver is a site where immune signals derived from the gut interact with the systemic immune system. It is at the juncture of the peripheral circulation and the portal circulation leading to interaction between naive T cells and hepatic cells. This interaction results in the generation or disruption of the development of tolerance to commensal bacteria and other environmental agents.^78^ The liver–gut crosstalk is the basis for the hepatobiliary manifestations of IBD.^79^ Gut microbiota are associated with the development of intestinal, hepatic and extra-intestinal manifestations of IBD.^80^ The pathogenesis of the liver manifestation of IBD is related to gut inflammation that results in inflamed portal tracts of the enterohepatic circulation of lymphocytes from the gut to the liver.^79^ It involves multiple gut-derived inflammatory cell types and cytokines, chemokines and other molecules that lead to the destruction of normal liver architecture.^78^ Both pathogenic and commensal microbiota trigger these events. Products of the microbiota activate the innate immune system to drive pro-inflammatory gene expression, inducing chronic inflammatory disease of the liver.^81^ The crosstalk has a role in the pathogenesis and outcome of primary sclerosing cholangitis and in immunoglobulin G4-associated cholangitis in patients with IBD.

Reduced intestinal availability of bile salts reduces stimulation of the farnesoid X receptor, inducing bile salt overload and hepatotoxicity through reduced action of intestinal fibroblast growth factor 19.^80^ Enteral lipids reduce inflammation and liver damage during stress or systemic inflammation, whereas parenteral lipid is associated with liver damage.^80^ CD8^+^ cells primed in the GALT acquire effector function and can migrate to the liver, leading to cholangitis in an antigen-dependent manner.^82^

## Targeting the gut immune system as a means for altering the systemic immune system for the treatment of IBD

**Figure 2:**
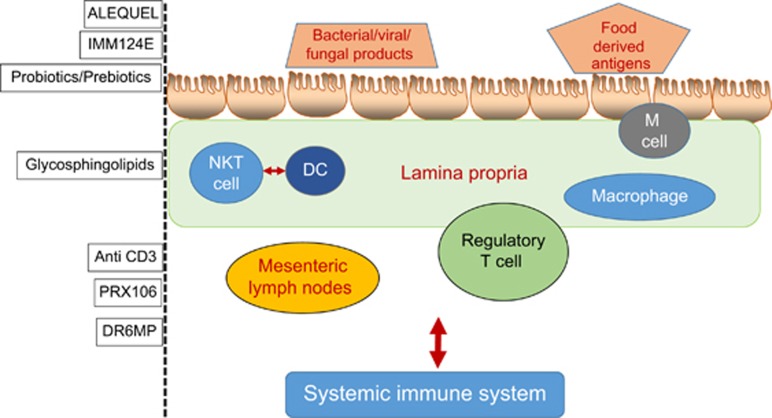
Several potential targets for oral immune therapy in inflammatory bowel disease at different levels of the immune system of the gut.

Diverse regulatory mechanisms cooperate to maintain intestinal homeostasis, and a breakdown in these pathways precipitates the chronic inflammatory pathology in IBD.^83^ Orally administered agents that alter the gut immune system and/or target the signals between the gut and the systemic immune systems can serve as means for altering the systemic immune system for the treatment of IBD. Described below are several examples of compounds being developed for oral immune therapy in IBD, some of which have been tested in humans (Figures 1 and 2).

## Oral administration of an extract of autologous colonic protein-derived antigens, Alequel

Oral immune therapy based on the oral administration of extracts of colonic proteins alleviated immune-mediated colitis in animal models of IBD.^45^ A marked reduction in the fraction of injured colonic areas and colon weights were observed, along with reductions in the inflammatory response and mucosal ulcerations. These effects were associated with an increase in TGFβ1 and a decrease in IFN-γ serum levels. TNBS-induced colitis was attenuated in naive recipients of splenocytes from tolerized rats compared with rats that received splenocytes from control donors.^45^

In humans, oral administration of Alequel, an extract of autologous colonic-derived proteins, was safe in patients with CD.^84^ In a phase I trial, 10 CD patients were orally treated with Alequel for 16 weeks. Seven patients achieved clinical remission and improvement in their IBD questionnaire scores. The high levels of colitis-extracted protein-specific IFN-γ-spot-forming colonies decreased with therapy. The beneficial effect was associated with alteration of the CD4^+^/CD8^+^ lymphocyte ratio, increased peripheral NKT cell numbers and increased serum IL-10 and IL-4 levels.^84^ In a phase II randomized, double-blind, placebo-controlled trial, 31 patients with moderate-to-severe CD were enrolled in a 27-week study.^85^ Clinical remission was achieved in 58% of the patients in the treated group compared with 29% in the placebo group. A clinical response was observed in 67% and 43% of the treated vs placebo groups, respectively. An improved IBD questionnaire score was observed in 43% of the treated vs 12% of placebo groups. A decrease in the number of subject-specific, antigen-directed-IFN-γ spot-forming colonies and an increased percentage of peripheral blood NKT cells were observed in drug-treated patients who achieved remission. In a subsequent phase II trial, 43 CD patients were enrolled in a randomized, placebo-controlled, double-blind trial. Remission was achieved in 43% of treated versus 33% of the placebo group in weeks 6–9. For weeks 9–12, the remission rate was 50% in the drug-treated vs 33% for the placebo groups. Altered NKT cell numbers and CD4^+^/CD8^+^ T-lymphocyte ratios were noted in treated patients. No treatment-related adverse events were noted in the studies.^86^ Alterations in CD4^+^, CD8^+^ and NKT lymphocytes cells support the notion that oral immune therapy using non-absorbable autologous proteins affects the systemic immune system.

The long-term learning ability of the gut immune system was recently published in these patients. A total of 92 patients treated with Alequel were followed. In patients who responded, the mean disease-free interval was 7.3±3.96 months. The opposite effect was noted for patients who received placebos.^87^ These results suggested that short-term oral administration of autologous colonic extracts exerts a long-term beneficial memory effect in moderate-to-severe CD.^87^ The long-term effect was associated with the promotion of regulatory/suppressor cells with memory phenotypes.

Taken together, the results suggest that oral immune therapy of non-absorbable Alequel is safe and effective for the treatment of moderate-to-severe CD. It induces a long-term beneficial memory effect in these patients.

## Oral administration of non-absorbable delayed-release 6-mercaptopurine in patients with CD

The purine analogs azathioprine and 6-mercaptopurine (6-MP) are a mainstay of long-term maintenance therapy for IBD patients.^88^ Their use is somewhat limited by systemic side effects that are associated with low tolerability and low compliance.^89, 90, 91, 92^ Adverse events are associated with discontinuation or dose reduction of therapy in 9–28% of patients.^90, 91, 92^ Serious adverse events were reported in 14% of patients receiving azathioprine. Dosing strategies to improve therapeutic response and reduce adverse reactions are being used.^93^

A novel formulation of low-dose, delayed-release 6-mercaptopurine (DR-6MP) was developed for oral immune therapy.^94^ Pharmacokinetic and proof-of-concept open-label studies showed that DR-6MP is not absorbed significantly. Administration of a single dose of DR-6MP increased systemic CD4^+^CD25^+^Foxp3^+^ Tregs 24 h after ingestion. In a phase I proof-of-concept trial, administration of DR-6MP in active CD patients for 12 weeks resulted in remission in 3 out of 10 vs one out of three patients treated with Purinethol.^94^ Seventy patients with moderately active CD were enrolled in a 12-week double-blind controlled phase II trial compared with Purinethol. DR-6MP had similar efficacy to Purinethol following 12 weeks of treatment. However, the time to maximal clinical response was 8 weeks for DR-6MP vs 12 weeks for Purinethol. A higher proportion of patients on DR-6MP achieved clinical remission at week 8 and showed improvements in IBD questionnaire score. DR-6MP led to a decrease of CD62^+^ expression in T cells, implying a reduction of lymphocyte adhesion to the site of inflammation. DR-6MP was safer than Purinethol, with significantly fewer adverse events. There was no evidence of drug-induced leukopenia in the DR-6MP group, and a much lower proportion of hepatotoxicity.^94^

The data suggest that oral immune therapy using a non-absorbable DR-6MP formulation is safe and biologically active in the gut. It is clinically effective, exerting a systemic immune response with low systemic bioavailability and a low incidence of side effects. A possible effect of absorbed metabolites was not ruled out in the above studies. However, the lack of an effect on the number of peripheral blood leukocytes supports a local action on the gut immune system that led to alteration of the systemic immune system documented by changes in CD62^+^ T cells.

## Oral administration of non-absorbable anti-CD3 antibodies in humans and in an animal model of colitis

Intravenous administration of anti-CD3 antibodies was shown effective in the transplantation setting and in several immune-mediated disorders owing to their ability to induce tolerance.^95^ In mouse models of autoimmune diabetes, parenteral administration of anti-CD3 antibodies induced disease remission by restoring tolerance to pancreatic β-cells in part of the treated animals or patients. These antibodies arrest ongoing disease by clearing pathogenic T cells from target organs. In humans with recently diagnosed diabetes, preservation of β-cell function was achieved by short-term administration of a CD3-specific antibody.^95^ Clinical trials using two distinct humanized Fc-mutated antibodies to human CD3, ChAglyCD3 (otelixizumab) and OKT3-γ-1 Ala-Ala (teplizumab), demonstrated that parenteral CD3 antibodies preserved β-cell function, maintaining high levels of endogenous insulin secretion in treated patients.^96^ Parenteral administration of visilizumab, a humanized low-Fc receptor binding anti-CD3 antibody, induced a rapid clinical response in patients with steroid-refractory ulcerative colitis. This antibody induced apoptosis of lamina propria CD4^+^ T cells isolated from non-IBD individuals, ulcerative colitis and CD patients.^97^ Incubation of the inflamed mucosal biopsy specimens from patients with IBD with otelixizumab reduced inflammation-associated tyrosine phosphoprotein of proteins associated with T-cell receptor activation.^98^ Encouraging results from phase 1/2 clinical trials have been reported for visilizumab and foralumab in patients with IBD.^99^ However, these parenteral CD3-based therapies have high rates of adverse events. Studies reveal a narrow therapeutic window of anti-CD3-based therapies, in which low doses are ineffective and higher pharmacologically active doses cause intolerable levels of adverse effects.

One of the goals for the immunotherapy of autoimmune diseases is the induction of Tregs that mediate tolerance while omitting generalized immune suppression. Oral administration of anti-CD3 has been tested as a means to promote Tregs via stimulation of the gut immune system which preferentially induces these cells.^100^ Orally delivered antibodies do not have side effects associated with generalized immune suppression, and do not induce cytokine release syndromes, making them clinically applicable both for chronic therapy and for preventive treatment.^101^

Orally administered anti-CD3 monoclonal antibody is biologically active in the gut and suppressed experimental autoimmune encephalomyelitis, an animal model of multiple sclerosis, in a dose-dependent fashion, both before disease induction and at the height of disease. Oral anti-CD3 antibody acts by inducing a unique type of Tregs characterized by latency-associated peptide (LAP) on its cell surface functioning via a TGF-β-dependent mechanism.^101, 102^ It suppressed the incidence of type 1 diabetes in an animal model via conversion of Th1 responses into Th2/Th3 in the periphery, including in pancreatic lymph nodes.^103^ Oral and nasal administration of anti-CD3 to a NZB and SNF-1 mouse model of lupus suppressed autoantibody production and prevented kidney damage.^104, 105^ Oral anti-CD3 alleviated type 2 diabetes and nonalcoholic steatohepatitis (NASH) in an animal model.^15^ Animals fed the anti-CD3 antibody with the gut adjuvant β-glucosylceramide (GC) showed reductions in pancreatic hyperplasia, hepatic fat accumulation and muscle inflammation; alleviation of type 2 diabetes; and reductions in liver enzymes and cholesterol and triglyceride levels.^15^ The effect of this type of oral immune therapy on the gut immune system was associated with the promotion of systemic CD4^+^LAP^+^ T cells, a decrease in NKT cells and an increase in TGF-β and IL-10 secretion from DCs and from anti-CD3-activated PBLs.

In mice with colitis, oral administration of anti-CD3 induced changes in the mucosal immune response that prevented colitis by affecting the systemic immune system.^106^ The effect was independent of a specific antigen and was associated with reduced T-cell activation in an IL-10-dependent manner. Oral anti-CD3 protected severe combined immunodeficient mice injected with CD4^+^CD45RBhigh T cells from colitis.^106^ No differences in total cell counts or percentages of CD4^+^ and forkhead box P3^+^ Tregs was noted between mice given anti-CD3 or controlled immunoglobulin. In mice with enteropathy, oral anti-CD3 reduced levels of inflammatory cytokines and increased levels of the anti-inflammatory cytokines IL-10 and TGF-β.^106^

In an open-label Phase I clinical trial comprising 18 healthy males, oral anti-CD3 was biologically active and well tolerated. No systemic treatment-related adverse effects were noted.^107^ Specifically, there was no change in the CD3^+^ lymphocyte count and no human anti-mouse antibodies were detected, implying non-absorption of the antibodies and a local effect on the gut immune system. The local effect was associated with an effect on the systemic immune system manifested by suppression of the Th1/Th17 and IFN-γ responses and increased CD4^+^CD25^+^ and CD8^+^CD25^+^ T cells.^107^ Treatment was associated with a decrease in IFN-γ and IL-17 and an increase in TGF-β secretion from anti-CD3-stimulated PBLs. In addition, decreased IL-23 and IL-6 expression and increased IL-10 and TGF expression in DCs, along with decreased IFN-γ and IL-17 secretion from anti-CD3-stimulated PBLs, and decreased IL-23 expression in DCs, were noted.^107^

In a Phase-IIa trial of patients with type 2 diabetes and NASH, oral administration of several dosages of anti-CD3 was biologically active and well tolerated without treatment-related adverse events. While exerting its effect on the gut immune system, it promoted Tregs systemically, manifested by an increase in CD4^+^LAP^+^ and CD4^+^CD25^+^LAP^+^ cells, with an increase in TGF-β serum levels. Decreases in plasma glucose and liver enzymes were noted in a dose-dependent manner.^108^

Taken together, the animal models and the human studies support an oral immune therapy effect on the systemic immune system for the non-absorbable anti-CD3. A lack of systemic absorption or an effect on leukocyte numbers supports the high safety profile of this method.

## Oral administration of non-absorbable anti-lipopolysaccharide antibodies with adjuvants in humans and in an animal model of colitis

The gut microbiome and bacteria-derived products are relevant to the pathogenesis of IBD and can serve as targets for oral immune therapy. Imm124E is an IgG-enriched fraction of enterotoxigenic *E*. *coli*-containing colostrum that contains anti-lipopolysaccharide and several glycosphingolipid adjuvants (Immuron, Melbourne, VIC, Australia). Induction of oral immune therapy using the non-absorbable Imm124E formulation altered the systemic immune system. In the ob/ob model of diabetes and NASH, oral administration of Imm124E decreased serum TNF-α levels, and increased the number of splenic CD4^+^CD25^+^, CD4^+^CD25^+^Foxp3^+^ Tregs, and NKT cells. The effects on the systemic immune system were associated with a decrease in ALT serum levels and hepatic triglycerides, and improved glucose intolerance.^109^

In humans, in an open-label trial, subjects with biopsy-proven NASH and type 2 diabetes were orally administered Imm124E^110^ for 30 days. No treatment-related adverse events were observed and no human anti-bovine antibodies were detected. Increased serum levels of GLP-1 and adiponectin, along with the promotion of CD25^+^ and CD4^+^CD25^+^Foxp3^+^ Tregs, were noted. These effects on the systemic immune system were associated with the alleviation of insulin resistance as determined by a decrease in fasting glucose levels, an increase in early insulin secretion following glucose administration, and improvements in the glucose tolerance test and HBA1C levels. Treated patients showed a decrease in the serum levels of triglycerides, total cholesterol and low-density lipoprotein cholesterol, and a decrease in liver enzymes.^110^

In the mouse-TNBS colitis model, oral administration of Imm124E increased serum levels of the anti-inflammatory cytokine IL-10 and promoted both CD4^+^CD25^+^ and CD4^+^Foxp3^+^ Tregs. These effects were associated with an amelioration of body weight loss and improved bowel histology. The extent of the disease, and the inflammation, colitis damage and regeneration scores decreased in treated mice.^111^

The animal models and the human studies suggest that oral immune therapy using non-absorbable Imm124E alters the immune-mediated clinical manifestations by exerting a local effect on the gut immune system. The lack of absorption supports the high safety profile of this immune modulatory method.

## Oral administration of non-absorbable glycosphingolipids in humans and in an animal model of colitis

Oral administration of β-glycosphingolipids has been tested as a means to alter NKT cells in immune-mediated disorders.^112^ Glucocerebroside (β-glucosylceramide, GC) and lactosylceramide are metabolic intermediates in the metabolic pathways of complex glycosphingolipids.^113^ Their immune modulatory effects were shown in several studies.^112^ GC inhibited NKT lymphocyte proliferation in the presence of DCs.^114^ It increased the peripheral/intrahepatic NKT lymphocyte ratio, decreased serum IFN-γ levels and increased serum IL-10 levels, exerting a beneficial immune modulatory effect alleviating inflammation in several animal models of immune-mediated disorders.^44, 112, 114^ In a leptin-deficient ob/ob mice model of diabetes and NASH, oral administration of GC altered NKT cell distribution and the cytokine profile in an anti-inflammatory direction.^115^ The effects were associated with a decrease in liver size and hepatic fat content, near-normalization of glucose tolerance and decreased serum triglyceride levels.^115^ A synergistic beneficial effect was noted for the combination of GC and lactosylceramide in animal models of diabetes and NASH.^116, 117^

In the TNBS colitis model, oral administration of GC led to an increased peripheral/intrahepatic CD4/CD8 lymphocyte ratio, decreased STAT-1 and -4 expression, and overexpression of STAT-6, along with decreased IFN-γ serum levels.^44^ The effects on the systemic immune system were associated with the alleviation of colitis manifested by improvement of both the macroscopic and microscopic scores.

In humans, oral administration of GC to patients with diabetes and NASH was tested in a randomized, double-blind placebo-controlled trial.^118^ No treatment-related adverse events were noted. An increase in peripheral NKT regulatory lymphocytes was observed. This effect was associated with a decrease in HBA1C levels, improvement in the glucose tolerance test, increased HDL levels and a decrease of hepatic fat by magnetic resonance imaging.^118^

The data suggest that non-absorbable glycosphingolipids exert an effect on the innate immune system of the gut thereby altering the systemic immune system.

## Oral administration of non-absorbable PRX106 in an animal model of colitis

Parenteral administration of etanercept is successfully used in the treatment of rheumatoid arthritis but is not effective for the treatment of CD.^119, 120, 121^ In some cases, it was associated with exacerbation of the disease.^122^ A different effect on lamina propria lymphocytes was suggested as a possible explanation.^123^

PRX-106 is a non-absorbable orally administered BY-2 plant cell that expresses a recombinant anti-TNF fusion protein that consists of the soluble form of the human TNF receptor fused to the Fc component of a human antibody IgG1 domain. PRX-106 has an amino acid sequence identical to etanercept. *In vitro,* PRX-106 binds TNF alpha, inhibiting it from binding to cellular TNF receptors and blocking its downstream effects.^124^ Oral administration of BY-2 plant cells expressing PRX-106 resulted in altered distribution of hepatic Tregs with a significant alteration in the distribution of CD4^+^CD25^+^FOXP3^+^ cells and in CD8^+^CD25^+^FOXP3^+^ cells. A change in the spleen/liver ratio for CD4^+^CD25^+^FOXP3^+^ was noted. The effects on the systemic immune system of the non-absorbable formulation were associated with alleviation of immune-mediated colitis in a mouse model.^124^ Improvements in weight loss and of bowel histology were noted in the PRX-106-treated mice. A reduction in I-IkB-alpha phosphorylation was noted in colon samples, indicating a lower level of apoptosis in the inflamed tissues.

The data support the notion that oral administration of a non-absorbable formulation of plant cells expressing recombinant anti-TNF fusion protein has biological activity in the gut, and can alter the systemic immune system to exert a beneficial immune modulatory clinical effect. It may provide a new, safer and more effective anti-TNF alpha-based immune therapy for IBD.^124^

## Oral administration of *Lactococcus lactis* secreting an anti-TNF nanobody in animal models of colitis

*Lactococcus lactis* is a lactic acid Gram-positive food-grade bacteria that is safely consumed. It was genetically engineered and orally formulated to deliver therapeutic proteins in the bowel for immune modulation.^125^ A tolerogenic live Lactococcus lactis bacteria was engineered for controlled secretion of the type 1 diabetes autoantigen GAD65370-575 and IL-10 in the gut. In combination with short-course low-dose anti-CD3, this treatment increased systemic CD4^+^Foxp3^+^CD25^+^ Tregs, improved insulitis and functional β-cell mass and restored glucose levels in recent-onset NOD mice.^126^ Similarly, *Lactococcus lactis* was engineered to secrete monovalent and bivalent murine (m)TNF-neutralizing nanobodies. Its oral administration resulted in local delivery of anti-mTNF nanobodies at the colon and significantly reduced inflammation in mice with dextran sulfate sodium-induced chronic colitis, and improved established enterocolitis in IL-10^−/−^ mice.^127^ The data supports the concept that systemic tolerogenic effects can be achieved via an effect on the gut immune system.

## Nutraceuticals, functional foods, prebiotics, probiotics, polyunsaturated fatty acids, amino acids and polyphenols, and other gut-associated adjuvants, can re-establish gut tolerance by altering the gut immune system and/or via modulation of intestinal microbiota

Amino acids (glutamine, arginine, tryptophan and citrulline), fatty acids (short-chain and omega-3 fatty acids and conjugated linoleic acids) and probiotics (Bifidobacterium, Saccharomyces and Lactobacillus) can restore the intestinal barrier, supporting gut barrier integrity and function.^128^ Probiotics prevent pathogen adherence and invasion of the epithelium by blocking adherence sites and upregulating the gene expression of MUC2 and antimicrobial peptides.^129^ Probiotics restore eubiosis and potentially restore the deleterious effects of bacterial metabolites and of unabsorbed dietary constituents with the production of free radicals and phenols associated with cell damage. Probiotics affect the innate inflammatory response of epithelial cells to stimuli from the gut lumen reducing inflammation. They exert an effect on DCs and on epithelial cells to affect naive T lymphocytes in the lamina propria, thereby affecting adaptive immunity.^129, 130^

In both CD and ulcerative colitis, the advantage of probiotics remains unproven.^131^ Some studies, however, did show a beneficial effect. *E. coli* Nissle 1917 maintained remission, suggesting that it can serve as an alternative in patients intolerant or resistant to 5-aminosalicylic acid preparations. In pouchitis, small controlled trials suggest a benefit from VSL no. 3 in the primary and secondary prevention of pouchitis.^131^ Taken together, the data support the possibility that probiotics and prebiotics may alter the systemic immune system via a gut signal.

## Summary

Oral immune therapy may overcome many of the obstacles of the agents currently used in the treatment of IBD. Oral immune therapy methods are based on the exertion of an effect on the gut innate immune system and/or on the microbiome, thereby inducing an immune signal that can alter the systemic immune system. Oral immune therapy provides a means for immune modulation with minimal side effects, as it does not involve generalized immune suppression. While overcoming the adverse events barrier, large clinical trials are required to show the efficacy of these treatments in IBD. In the future, this type of immune modulation may present an alternative to the currently used immune suppressive and immune modulatory agents.

## Figures and Tables

**Table 1:** Advantages of oral immune therapy for the treatment of inflammatory bowel disease in contrast with the disadvantages of systemic immunomodulatory agents

	*Oral immune therapy*	*Systemic immune modulators*
Mechanism	Takes advantage of the inherent ability of the gut's immune system to control unwanted systemic immune responses	Act from the outside of inflammatory pathways
Generalized immune suppression	Not associated with general immune suppression	Induces generalized immune suppression
Induction of regulatory T cells	Preferentially induces regulatory T cells	May reduce regulatory T cells
Induction of tolerance	Can induce systemic tolerance	Does not induce tolerance
Target antigen dependency	Can be induced in an antigen-dependent or -independent manner	Not antigen-dependent
Reach the blood	Most compounds used do not reach the blood system	Needs to reach the blood
Toxicity	Minimal side effects	Significant toxicity including risks of infection and malignancy. Toxicity and side effects limit their use in a major proportion of patients
Cytokine release syndrome	Not associated with a harmful cytokine release syndrome	May be associated with cytokine release syndrome
Prevention or treatment	Effective both for preventive therapy and for treatment at the peak of disease	Many of the compounds used are effective for an established disease, and due to potential toxicity, they are not ideal for prevention.
Maintenance therapy	Can be used for maintenance	For several compounds, the toxicity prohibits their use for maintenance therapy
Dose	No absorption is required; therefore, a relatively low dose is sufficient for achievement of a clinically meaningful effect	Relatively high dosages are required depending on bioavailability
Platform	A platform that can be used for many disorders	Some compounds are disease specific
Patient advantages	Easily tolerated	Toxicity may limit tolerability
Safety concerns and pain	Eliminates safety concerns and pain related to needles.	Safety concerns and pain related to use of needles may limit their use for some patients
Requirement for trained personnel	Trained medical personnel not required for administration	For some compounds, intravenous or subcutaneous administration along with trained personnel are required
Cost	Relatively low cost	Expensive
